# Shape-Stabilized PEGylated Silica Aerogel-Composite as an Energy Saving Building Material

**DOI:** 10.1021/acs.iecr.3c02373

**Published:** 2023-11-15

**Authors:** Adeel Arshad, Anurag Roy, Tapas K. Mallick, Asif Ali Tahir

**Affiliations:** Solar Energy Research Group, Environment and Sustainability Institute, University of Exeter, Penryn Campus, Cornwall TR10 9FE, U.K.

## Abstract

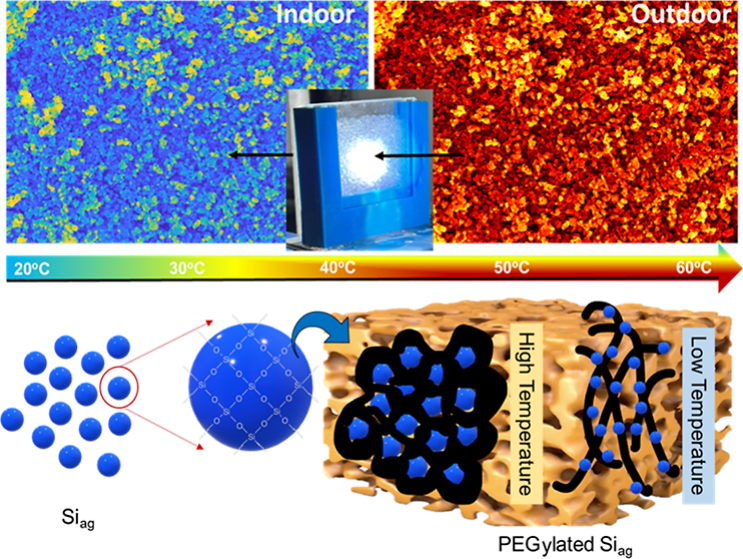

Balancing thermal and visual comfort in buildings necessitates effective insulation to counteract heat loss and gain, especially with temperature variances. One promising approach is to combine phase change materials, such as poly(ethylene glycol) (PEG), with high-performance insulators like silica aerogel (Si_ag_). To bolster opto-thermal performance in building envelopes, we introduce a smart insulation composite material through PEG integration, i.e., PEGalyation with Si_ag_. Central to this thermal behavior is the PEG’s phase change properties, which foster a shape-stabilized framework with Si_ag_ through their porous confinement. Preliminary observations indicate notable capabilities of obstructing near-infrared light while preserving satisfactory visible transparency. An optimized Si_ag_@PEG composite with 5% loading of PEG has the visible range transmission of ∼92%, a decrease of ∼72% in thermal conductivity which is lower than pure glass and PEG, leading to a temperature dependent switchable hydrophobic to hydrophilic wettability characteristics. As a prototype window, the thermal performance evaluation of the synthesized composite, through experimental and computational studies, shows a decrease in indoor temperature of ∼20% with a higher temperature difference of ∼20 °C between outdoor and indoor weather conditions. This lightweight composite can act as sponge media to fill inside the double-paned window and for retrofitting existing glazing to boost the energy efficiency of buildings with facile manufacturing and scalability.

## 1 Introduction

Improving the energy efficiency of UK buildings is the most immediate solution to aid families and businesses in countering the surge in energy prices. This not only offers health benefits but also enhances indoor living conditions and curtails energy bills.^1^ Retrofitting building envelopes, particularly windows, can significantly reduce the energy consumption. Wasted energy accounts for a significant proportion of windows, ranging from 20 to 40% in a building. Space heating and cooling currently consume 20% of a building’s total energy, and this number is projected to rise to 50% by 2050.^2,3^

While windows play a crucial role in maintaining the connection between the internal and external environment, they contribute significantly to the overall energy consumption of a building due to their transparent nature, which allows heat loss, gain, and daylight.^4^ Enhancing solar heat energy technology is a promising approach to decreasing the cooling load demand caused by solar gain penetrating a building through a transparent façade. Consequently, reducing energy consumption through energy-efficient building envelopes is critical for achieving energy-hungry buildings. In this scenario, the development of smart windows is essential for managing energy consumption in buildings, especially given the rising energy costs attributed to climate change.

Transparent insulation materials (TIMs) are advanced materials that can capture and retain solar heat energy by reducing heat losses.^5^ They improve the insulation capacity by minimizing heat energy flow within small air gaps or evacuated spaces in low thermal conductivity materials. Thermal insulation is the most attractive option for improving windows’ energy efficiency, with aerogel being the most potent component that can be used in building materials. Silica aerogel, with its low thermal conductivity and high optical transmittance, is a potential candidate for energy-saving window materials.^6,7^ The ability of TIMs to reduce thermal loss and provide solar transmission is dependent on the operating temperature, geometrical structure, and material types. Therefore, developing smart windows that focus on thermal insulation and transparency is an effective strategy to decrease energy consumption in buildings.^8,9^

However, the effectiveness of composite-based TIMs with polymers,^10^ phase change materials,^11,12^ or metal oxides^13^ in reducing thermal loss and providing solar transmission is influenced by factors such as operating temperature, geometrical structure, and material types.^14^ Silica aerogels are solid materials synthesized through a complex sol–gel process that involves precursor preparation, gelation, aging, surface modification, and drying. While these materials exhibit remarkable insulation properties, their inherent brittleness limits their practical applications, making their processing and handling complicated. Incorporating fibers into the aerogel structure has been shown to be an effective strategy for overcoming this limitation, leading to the production of aerogel composites that have a wider range of potential applications.

Researchers have explored different silica aerogel-polymer composites that use silica aerogels as fillers in a polymer matrix. Organic phase-change materials (PCMs),^15^ such as eicosane, erythritol, and paraffin, have been embedded in silica aerogel powders to improve the mechanical and thermal properties of the material.^16−18^ SiBNO/SiO_2_ aerogel composites have been demonstrated to have potential for use in aerospace and antenna windows due to their high solar transmittance efficiency and excellent heat insulation properties.^19^ Similarly, TiO_2_@SiO_2_ in a polyurethane matrix-based composite has been shown to produce a transparent coating with excellent thermal insulation and UV-shielding properties.^20^ A hollow SiO_2_ and indium tin oxide nanoparticle composite with poly(ether sulfone) also shows promise, with low thermal conductivity and high near-infrared shielding, as well as high visible transparency.^21^ Epoxy resin dispersion containing tungsten-doped vanadium dioxide nanoparticles effectively reduces indoor temperature, while VO_2_-polymer composite materials exhibit temperature-responsive color variation and enhanced IR modulation.^22^ A Cs_*x*_WO_3_–ZnO–SiO_2_ composite effectively shields most ultraviolet light and inhibits photochromism.^23^ Thermal insulation plays a pivotal role in achieving thermal comfort for building occupants by minimizing unwanted heat loss and gain. Concurrently, it is crucial to ensure that heat does not escape to the outdoors, especially during cooler nights or when the external temperature is lower than that of the interior. Balancing thermal insulation with the self-production of heat is challenging and often requires integration of multiple materials or systems with the building. This not only complicates manufacturing but also increases the costs and maintenance risks. A plausible solution could involve utilizing phase change materials, such as PEG, which has a critical solution temperature close to ambient. When integrated with a high-performance thermal insulator such as silica aerogel, PEG can form a synergistic system that optimizes energy efficiency in built environments at a reasonable cost. The methodology employed for the incorporation of PEG within porous matrices relies on capillary action, rendering it amenable to upscale production.^24,25^ The common results revealed that the embedded PEG localized within micro- and mesopores behaves akin to a nanofluid. This highlights that the thermal dynamics of nanoconfined PEG diverges from that of its bulk counterpart, an attribute attributed to the porous confinement. Fang Tian and colleagues discovered that PEG molecules in silica gel’s small pores had reduced energy and delayed temperature phase changes due to incomplete crystallization.^26^

To create a bifunctional smart window for both thermal insulation and self-production of heat, a PEGylation strategy was introduced using silica aerogel.^25,27,28^ PEGylation involves modifying silica aerogel with poly(ethylene glycol) (Si_ag_@PEG) using an economically viable manufacturing route and without supercritical drying process. The PEGylation process affects the micro- and mesopores of the aerogel, which may be affected on the surface or trapped inside the silica aerogel using filtration. The Si_ag_@PEG composite was easy to develop without toxic chemicals and complicated processes, such as supercritical drying and high-temperature heating. The synthesized composite’s thermal camouflage performance was examined at different temperatures, followed by its performance evaluation for buildings’ indoor thermal comfort, with Raman spectra, surface area analysis, and morphology analysis being used for optical and structural analysis of the Si_ag_@PEG samples. The porous composite matrices serve as numerous stand-alone storage units to store large amounts of PEG, and the formed composites possess a higher heat transfer efficiency than a large chunk of PEG. Additionally, the structural framework of these porous matrices augments the storage fidelity of PEG, attributable to the intricate interplay of hydrogen bond dynamics, capillary forces, wettability, and density phenomena. The primary objective was to assess the energy-saving capabilities of the Si_ag_@PEG composite in comparison to conventional double-glazed units.

## 2 Materials and Methods

### 2.1 Materials

Tetramethyl orthosilicate (TMOS), ammonium hydroxide (NH_4_OH), methanol (HPLC grade), and pentane were purchased from Merck UK Life Sciences. Polyethylene glycol (PEG, MW = 20 k), acetone, and ethanol were purchased from Alfa Aesar, UK. Bayer Corporation provided the diisocyanate cross-linker (Desmodor N3200). All the chemicals were used without further purification.

### 2.2 Synthesis of Silica Aerogel

A monolithic silica hydrogel, cross-linked with diisocyanate, was prepared from tetramethyl orthosilicate (TMOS) via a base-catalyzed route, following a published procedure.^29^ Notably, the gel was dried under ambient pressure without the use of supercritical fluid extraction. Initially, a solution of 0.1 M TMOS was prepared by dissolving it in a methanol/water mixture (1:1) under constant stirring. Then, 0.1 M NH_4_OH was slowly added dropwise to the solution until the pH reached ∼8.5, and the solution was aged for 48 h to obtain the silica gel (alcogel). Subsequently, the aged gel was washed repeatedly with ethanol and water. To cross-link the gel, diisocyanate cross-linker was dissolved in acetone and added to the aged gel, which was further aged for 24 h. Afterward, the gel was kept in an oven at 55 °C for 2 days. Finally, the dried gel was washed with acetone several times, followed by solvent exchange with pentane, and then dried at room temperature. The synthesized gel was ground with a mortar pestle and used for further analysis.

### 2.3 Preparation of PEGylated Silica Aerogel Composite

A composite of PEGylated silica aerogel was synthesized via a sol–gel process, followed by freeze-drying. During the aging process, a microlevel phenomenon called Ostwald ripening occurred, whereby small particles with unreacted OH moieties dissolved and reprecipitated into larger particles or condensed into more favorable regions of secondary particles, such as pores or crevices.^30^ To prevent the shrinkage of the aerogel’s skeleton, the solvent was rapidly frozen using liquid nitrogen and then sublimated under vacuum, resulting in highly porous aerogels.

**Figure 1:**
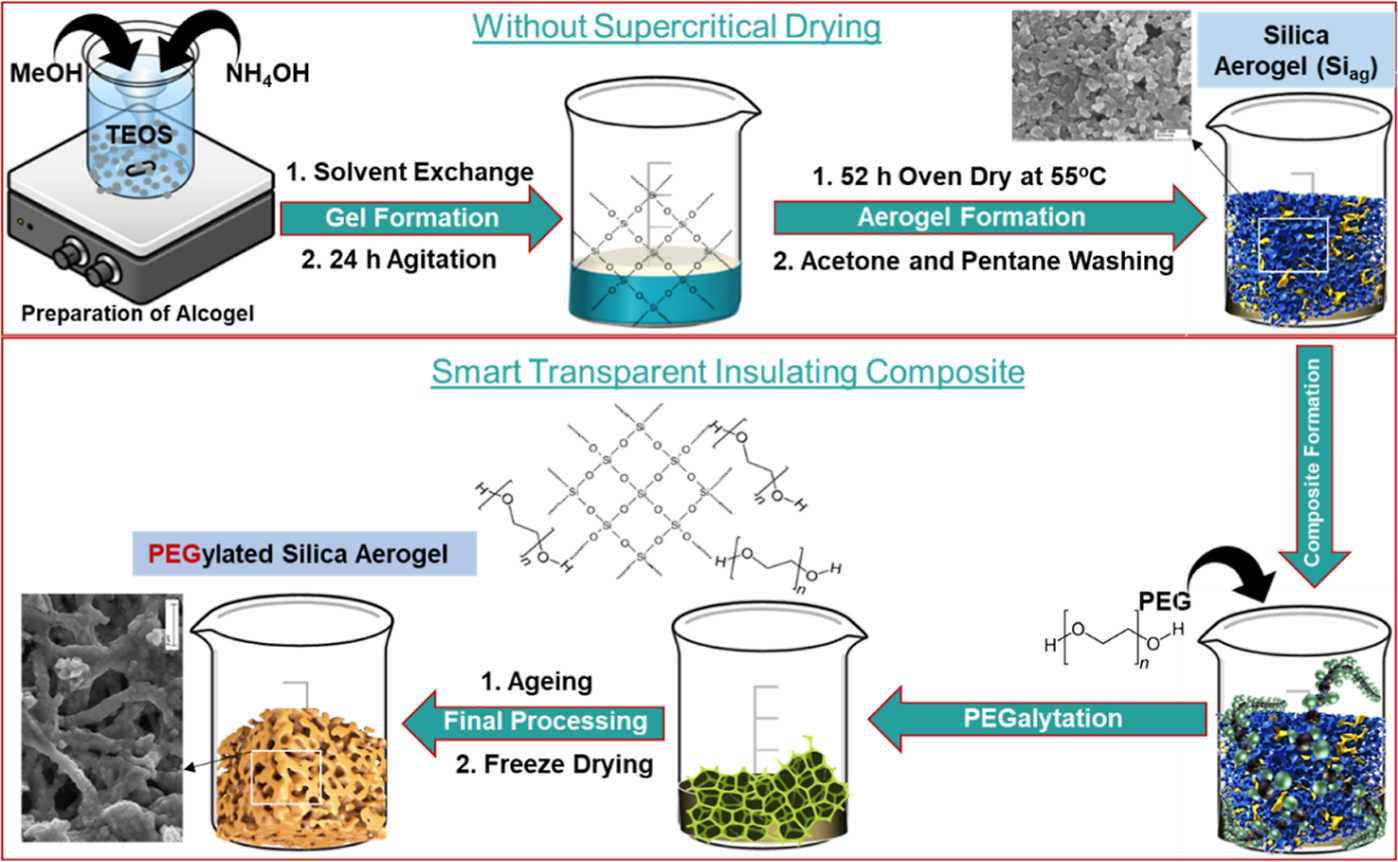
Schematic representation of the silica aerogel preparation without supercritical temperature, followed by the composite development through PEGylation method.

To synthesize the Si_ag_@PEG composite, silica aerogels were first synthesized and then mixed with PEG at different weight percentages (w/w %) under constant stirring at 60 °C for 2 h to obtain a homogeneous Si_ag_@PEG sol. The resulting sol was then cast into cylindrical molds and left to gel, forming Si_ag_@PEG hydrogels. The hydrogels were then frozen in liquid nitrogen and dried in a freeze-dryer for ice sublimation. After 48 h in the lyophilizer, the samples were collected and heated under a nitrogen atmosphere at 100 °C for 1 h to obtain the final PEGSi_ag_ composite sample. The composite samples with 3, 5, and 10% (w/w %) loading weight of silica aerogels were designated as Si_ag_@PEG-3%, Si_ag_@PEG-5%, and Si_ag_@PEG-10%, respectively. A schematic representation of the Si_ag_@PEG composite synthesis process is shown in Figure 1.

### 2.4 Material Characterization

The transmittance spectra of Si_ag_@PEG samples were obtained within the wavelength range of 200 to 2000 nm using a LAMBDA 1050 spectrophotometer (PerkinElmer).^31^ Temperature-dependent spectrophotometry measurements were performed by providing heat at different temperatures through a thermocouple. The impact of the temperature was investigated solely for the Si_ag_@PEG sample. The microstructural analysis of Si_ag_@PEG samples treated at various temperatures was conducted by using a FEI Quanta FEG 650 scanning electron microscope (SEM). The SEM images underwent additional analysis using MIPAR Image Analysis software. A JEOL 2100 instrument (200 kV) was used to capture images through Transmission Electron Microscopy (TEM). The hydrogel’s Raman spectrum and mapping were recorded utilizing a WITec Alpha 300R. To simulate 1 sun condition with an approximate light intensity of 100 mW/cm^2^, a Newport 66902, 300 W xenon lamp with an air mass (AM) of 1.5 was employed. The Pico Technology TC-08 thermocouple data logger was utilized to record indoor and outdoor temperatures. The IR images were captured at a distance of 10 mm using a FLIR T425 camera and processed with appropriate software to generate final images.^32^ Nitrogen physisorption measurements were performed using a Quantachrome (iQ3) instrument after evacuation at 100 °C for 2 h. The specific surface area was calculated by using the BET method, whereas the desorption cumulative pore volume and pore size distribution were determined by using the BJH method. Porosity measurements were conducted through Mercury Porosimetry Analyzer using the Quantacore Instrument, USA (Model PM 60-GT-16). The surface wettability of the composite films was accomplished by measuring the successive water contact angles on a drop shape analyzer (Kruss DSA25) using Young’s equation (sessile drop method). The volume of each drop was fixed at 5 μL and the dosing rate was 500 μL min^–1^. The instrument was equipped with a CCD camera for image capture. Thermal conductivity and heat capacity measurements for all samples were conducted utilizing a NETZSCH LFA-467 Hyperflash apparatus. The density measurement was performed on Brookfield Ametek DVNext equipment. Thermogravimetric analysis (TG) was conducted using a PerkinElmer Diamond TG/DrTG. Thermogravimetric/Differential Thermal Analyzer from 30 to 700 °C with a heating rate of 10 °C/min under nitrogen atmosphere. The temperature profile of the window prototype chamber, which contains the composite, was assessed under different solar intensities by using a Wacom AAA + continuous solar simulator (model WXS-210S-20).

## 3 Results and Discussion

### 3.1 Optical Transmission Characteristics

**Figure 2:**
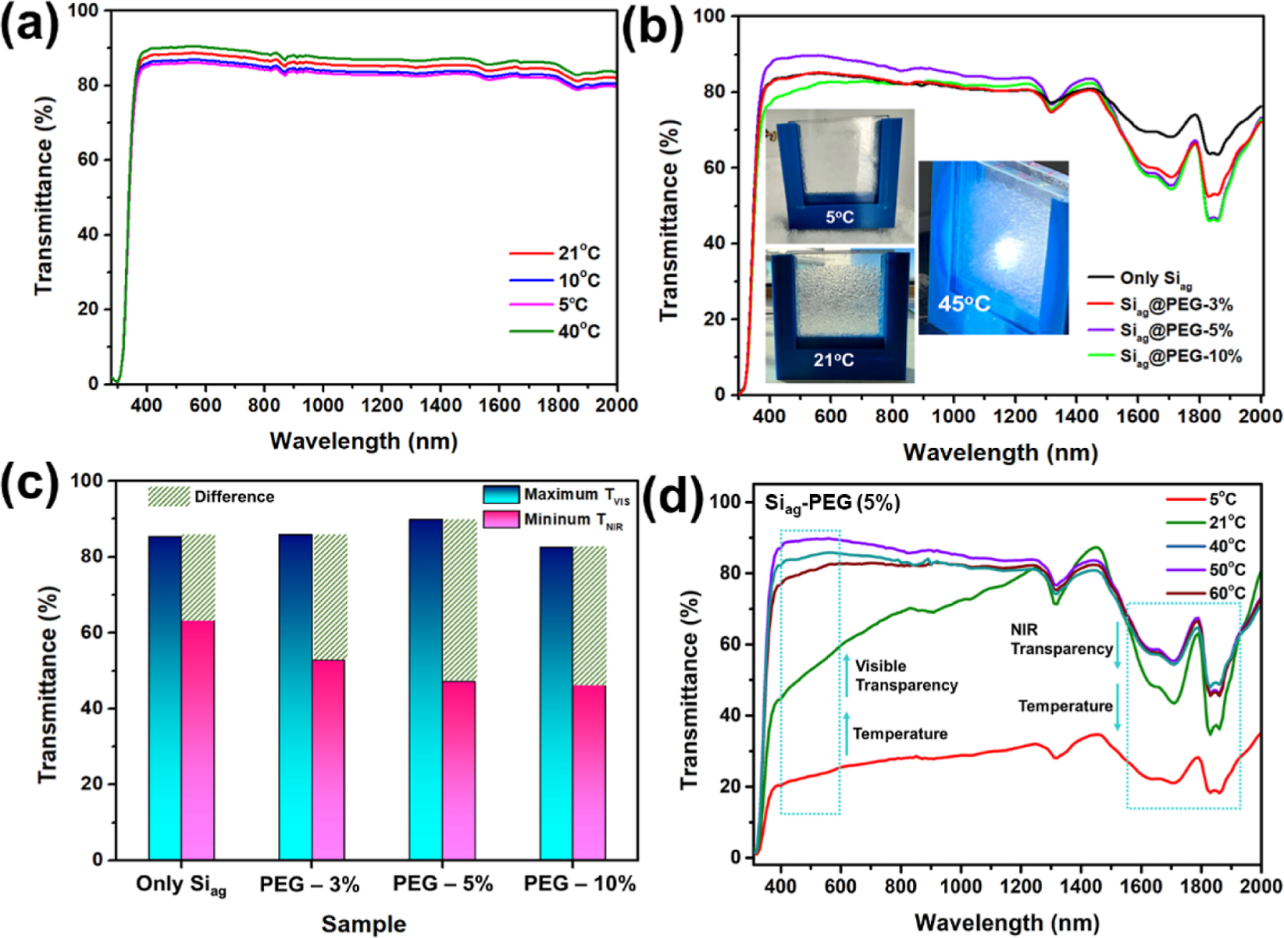
(a) The transmission spectra of the PEG sample were measured at various temperatures. (b) The transmission spectra of the Si_ag_@PEG composite samples were compared with those of the synthesized silica aerogel, and the inset shows photographs of the composite sample loaded into a prototype window at different temperatures. (c) A plot was created to show the temperature-dependent maximum visible transmittance and minimum NIR transmittance recorded for the Sia_g_@PEG composite samples compared to those of synthesized silica aerogel. (d) The transmission spectra of the Si_ag_@PEG-5% sample were recorded at different temperatures.

Figure 2a demonstrates that PEG exhibits high transparency (>80%) within the 200–2000 nm range, regardless of temperature. In contrast, the Si_ag_ sample synthesized in this study displays a maximum visible transmittance (*T*_VIS_) of 85.26% at 620 nm and a minimum near-infrared transmittance (*T*_NIR_) of 63.85% at 1850 nm at 22 °C. The transparency of Si_ag_ is significantly influenced by PEG incorporation, as illustrated in Figure 2b. The results indicate that increasing the quantity of PEG in the composite results in greater NIR blocking while maintaining a high *T*_VIS_. Notably, the Si_ag_@PEG-5% sample demonstrates enhanced *T*_VIS_ (∼90%), a 5% increase compared to only Si_ag_ and a 10% increase compared to Si_ag_@PEG-10%. The NIR transparency of Si_ag_@PEG-5% is comparable to that of Si_ag_@PEG-10%, while the *T*_NIR_ reduces by a maximum of 25%, as displayed in Figure 2a. Furthermore, Figure 2b displays real-time photographs of Si_ag_ exhibiting high transparency across the UV, visible, and NIR spectra. The greatest difference between *T*_VIS_ and *T*_NIR_ was found to be only Si_ag_ < Si_ag_@PEG-3% < 10% < 5%, as plotted in Figure 2c. Si_ag_@PEG-5% exhibits the greatest *T*_VIS_ and *T*_NIR_ differences and is thus selected for further analysis. The temperature dependence of the transmittance spectra for the PEG-5% sample is depicted in Figure 2d. At higher temperatures (>30 °C), the composite retains a higher maximum *T*_VIS_ at 50 °C. Whereas at 60 °C, *T*_VIS_ decreases by 15% compared to 50 °C. In contrast, *T*_NIR_ is not significantly affected. At lower temperatures (<30 °C), the composite displays a steady decline in *T*_VIS_ and less *T*_NIR_, similar to that observed at 21 °C. At 5 °C, *T*_VIS_ and *T*_NIR_ show similar values but are lower than 20%. The interdependence between *T*_VIS_ and *T*_NIR_ is contingent upon the temperature, where they exhibit a trade-off relationship. At low temperatures, the sample becomes semitransparent to opaque, whereas at higher temperatures, it remains highly transparent. These findings suggest that the composite can block more NIR light while maintaining high visible transparency at higher temperatures, providing a cooler thermal experience for warmer climates.^32^ Conversely, at lower temperatures, visible transparency is significantly impacted by PEG incorporation in the composite but retains higher *T*_NIR_, providing a warmer thermal experience for colder weather.

### 3.2 Raman Spectra, Surface Area, and Porosity Analysis

**Figure 3:**
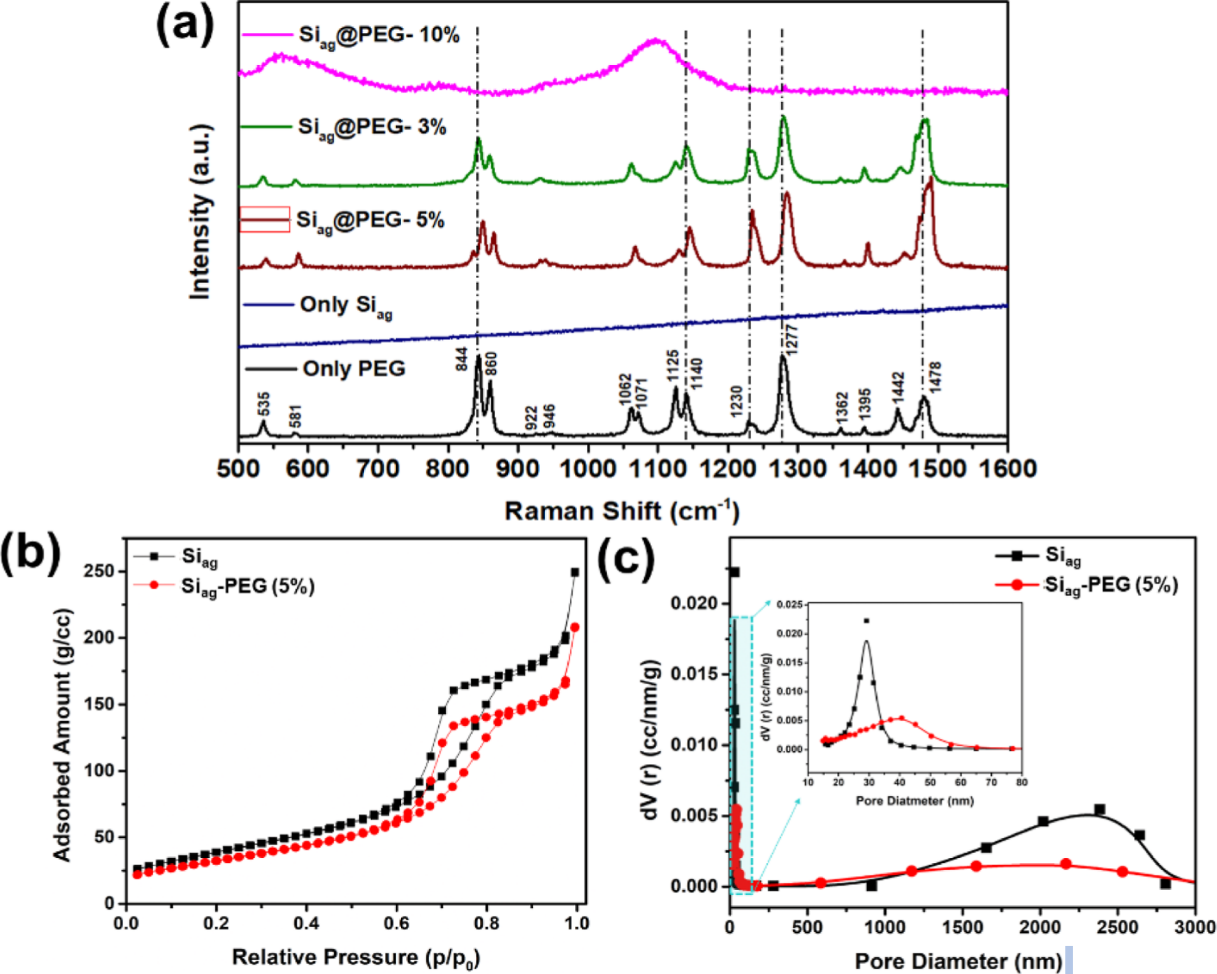
(a) Raman spectra of the Si_ag_@PEG composite samples compared with only Si_ag_ and PEG, (b) nitrogen absorption–desorption plot, and (c) Approximate macropore size distribution from the mercury intrusion branch only Si_ag_ and PEG@Si_ag_-5% samples, respectively.

The use of Raman spectroscopy can aid in comprehending the PEGylation technique for Si_ag_. Figure 3a exhibits the Raman spectra of the Si_ag_@PEG composite samples compared to those of their bare counterparts. The bands located at 1478 and 1442 cm^–1^ are assigned to the bending mode of the C–H group of the PEG, while the bands at 1277 and 1230 cm^–1^ correspond to C–H twisting vibrations.^33^ The C–O, C–O–H, and C–C stretching vibrations are located in the range of 922–1140 cm^–1^. The bands at 844 and 860 cm^–1^ are assigned to the skeletal vibrations of PEG. The positions at 581 and 535 cm^–1^ correspond to C–C–O bending vibration.^34^ However, no distinct Raman band is observed for the synthesized Si_ag_ sample.

In the case of the Si_ag_@PEG composite samples, the intensity of the composite Raman band has been reduced. This reduction was especially significant for Si_ag_@PEG-10% samples, which exhibited a combination of all characteristic peaks into a broadened one, indicating chemical compatibility between PEG and aerogels. The crystallinity of the PEG was significantly reduced due to the limited space for crystal growth. However, the Raman spectrum of Si_ag_@PEG-10% suggests that besides their physical change (amorphous), there is a possible morphological change that may occur during the preparation of PEG/aerogel powders due to the appearance of broader peaks. Moreover, PEGylation was conferred through labile C–O bonds via electrostatic interactions and surface functionalization. This interaction was most significant for Si_ag_@PEG-3%, which resulted in a shift to PEG’s characteristic Raman peaks. Additionally, the band shifts and intensity differences were considered to determine the average strength of the intermolecular hydrogen bond. Thus, strong composites were produced due to the Si–O–Si chemical bonds between the fibers and the silica matrix.

The Si_ag_@PEG-5% sample exhibited a specific surface area of 230 m^2^/g and had mesoporous characteristics similar to those of the synthesized Si_ag_ (256 m^2^/g), as observed from type IV isotherms in Figure 3b.

The pore size distribution of the composite was meticulously evaluated by using a mercury porosimeter. Distinct pore size distributions for both samples are evident, as illustrated in Figure 3c. The Si_ag_ sample manifests mesoporous characteristics, boasting a higher pore volume and an average pore diameter of 28 nm. In contrast, the Si_ag_@PEG-5% sample presents a broader mesoporosity, with an average pore diameter of 42 nm. Despite having a broader distribution, it possesses a reduced pore volume, relative to Si_ag_. This phenomenon can likely be attributed to the increased PEG content, which appears to enhance the dispersion of composite pores. However, the amplified PEG concentration seems to limit the pore volume expansion in comparison to composites with reduced PEG. Furthermore, there is an observed distribution of larger pores, falling within the macro size range of 1–3 μm. This could plausibly result from the interstitial framework gaps among the composite molecules. A notable trend is the decrement in both pore volume and diameter in tandem with the progression of PEGylation. When the matrix is infused with nanosilica, this additive augment the pore structure, subsequently diminishing the matrix’s porosity courtesy of its filling effect.^35^ As the PEG content escalates, the interface between the PEG and aerogel amplifies, stemming from the aerogel’s hydrophobicity. This engenders the formation of cracks and voids, leading to the emergence of larger pores.

A minor decrease in BET surface area hinted that PEG molecules were trapped in the aerogel.^25^ However, the retention of a similar surface area of the Si_ag_@PEG-5% sample suggests that the composite was formed during the surface interaction.

The adsorption curve showed an upward convex shape, indicating that PEGylation was driven by surface interaction rather than capillary condensation (infiltration).^36^ This type of pore is likely formed between agglomerated crystallites, where intercrystallite voids of Si_ag_ developed with randomly stacked PEG fibers. The appearance of a type H-4 hysteresis indicated a stacked-plane (slit)-like pore shape.^37^ Furthermore, given the absence of any chemical reaction during the filtration process, minimal perturbations in the pore structure are anticipated.

In TIMs, pores >4 mm are commonly used to impede convective heat transfer within the pores. Although increasing porosity (or gas volume) can lower the effective thermal conductivity of porous structures, including both the solid and gas phases, the solid phase typically exhibits a higher thermal conductivity than the gas phase.^38^ Thus, the effective thermal conductivity still tends to be higher than that of stationary air. Emerging as a trend in insulation technology, dynamic insulation materials offer a controllable thermal conductivity, where the Si_ag_@PEG composite can be used.^39^

### 3.3 Microstructural Analysis

**Figure 4:**
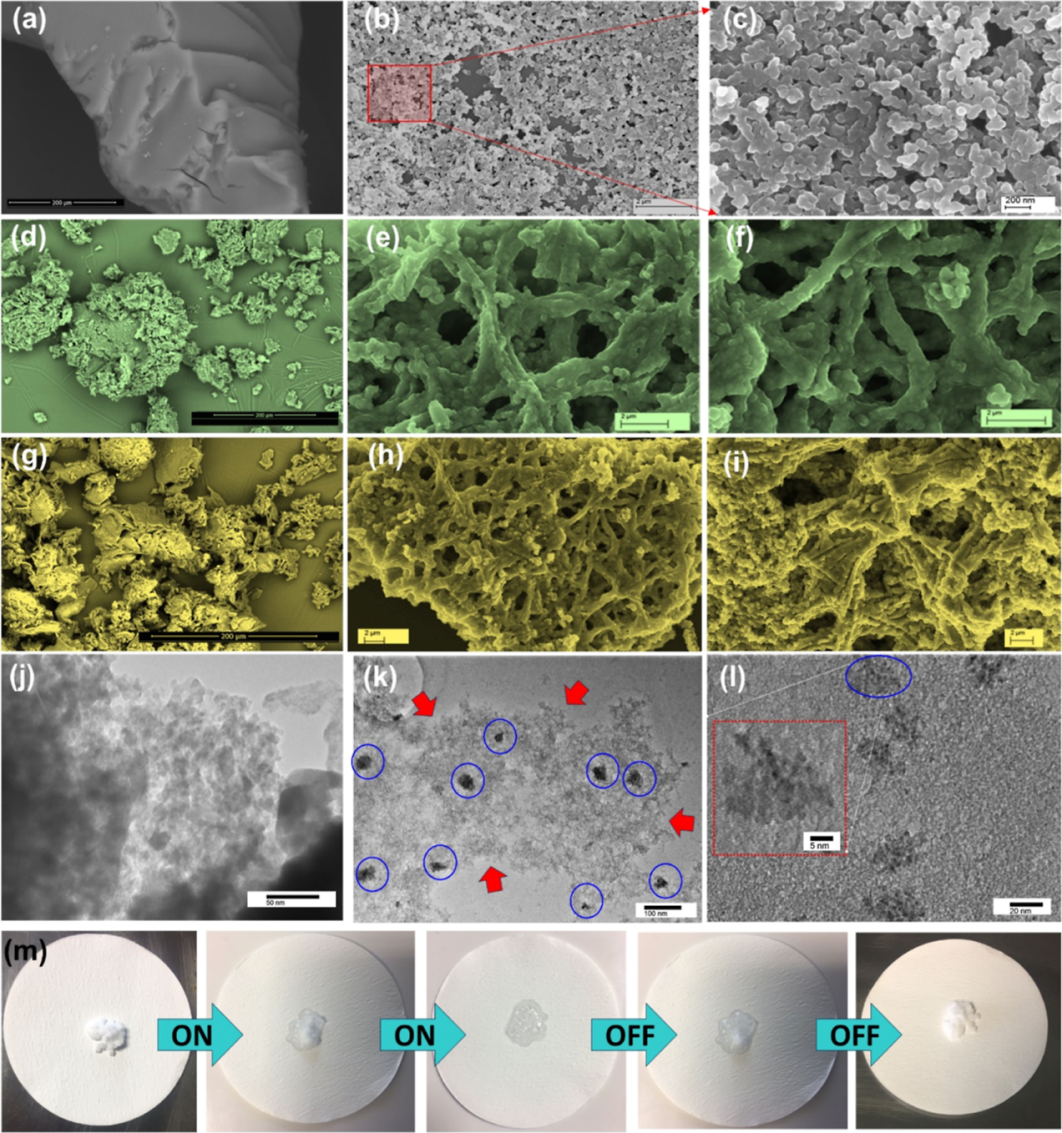
SEM micrographs of (a–c) only Si_ag_, (d–f) Si_ag_@PEG-5%, and (g–i) Si_ag_@PEG-10% samples at various magnifications, respectively. Bright-field TEM images of (j) only Si_ag_ and (k,l) Si_ag_@PEG-5% samples at various magnifications. In these images, red arrows point to the amorphous PEG network, while blue circles highlight the Si_ag_ embedded within the PEG network during the PEGylation process, (m) photographs of the Si_ag_@PEG-5% samples subjected to thermal treatment depict the composite’s shape-stabilizing properties without any evidence of leakage.

The measurements of surface area are of significant importance for the exploration of the microstructure of the composite. In Figure 4a–c, SEM micrographs of the synthesized Si_ag_ sample at different magnifications are presented, revealing a porous structure that is in good agreement with the density BET results. The particles display a uniform size with a diameter of <100 nm, indicating monodispersity. However, the morphology of the composite changes significantly upon PEGylation of Siag, resulting in a nanoparticle-embedded, interconnected porous network structure compared to Siag alone. The fibrous surfaces of the PEG network integrate randomly dispersed Siag nanoparticles, producing a highly entangled Si_ag_@PEG-5% sample, as shown in Figure 4d–f. The intrinsic entanglement is further increased by the higher inclusion of PEG in the Si_ag_@PEG-10% sample, as seen in Figure 4g–i. Moreover, the PEG fiber network in the Si_ag_@PEG-10% sample forms a dense assembly of closely packed nanoparticles, leading to a compact composite structure. Further investigation using bright-field TEM images reveals consistent structural orientations of Si_ag_ during the PEGylation process. Figure 4j showcases that the synthesized Si_ag_ exhibits an interconnected three-dimensional porous architecture. This structure is comprised of primary nanoparticles, approximately 30 nm in size, and clusters of aggregated nanoparticles. In contrast, Figure 4k,l exhibit that these Si_ag_ nanoparticles tend to agglomerate slightly during PEGylation, as indicated by the blue circles. Moreover, it is evident that during PEGylation, Si_ag_ becomes embedded within the PEG polymeric network (red arrows in Figure 4k), resulting in a composite that exhibits predominantly amorphous characteristics. The wrinkled channels prevented the leakage of PEG during the melting process, and the large pore volume of composite could benefit of PEGylation process. To visualize the shape-stabilizing property of the composite, Figure 4m displays photographs of the composite during heating and subsequent cooling. Upon heating, the composite melts, and when the heat is removed, it returns to its original form, corroborating its shape-stabilizing properties. In subsequent leakage experimental tests, the Si_ag_@PEG-10% composite (refer to Figure 4m) showed no signs of melted PEG on the filter paper. This is indicative of the successful synthesis of the composite and its ability to maintain shape stability during thermal treatments. These findings underscore the need to further evaluate their potential as energy-efficient building material.

### 3.4 Composite’s Thermal Performance Evaluation through a Prototype Window Model

**Figure 5:**
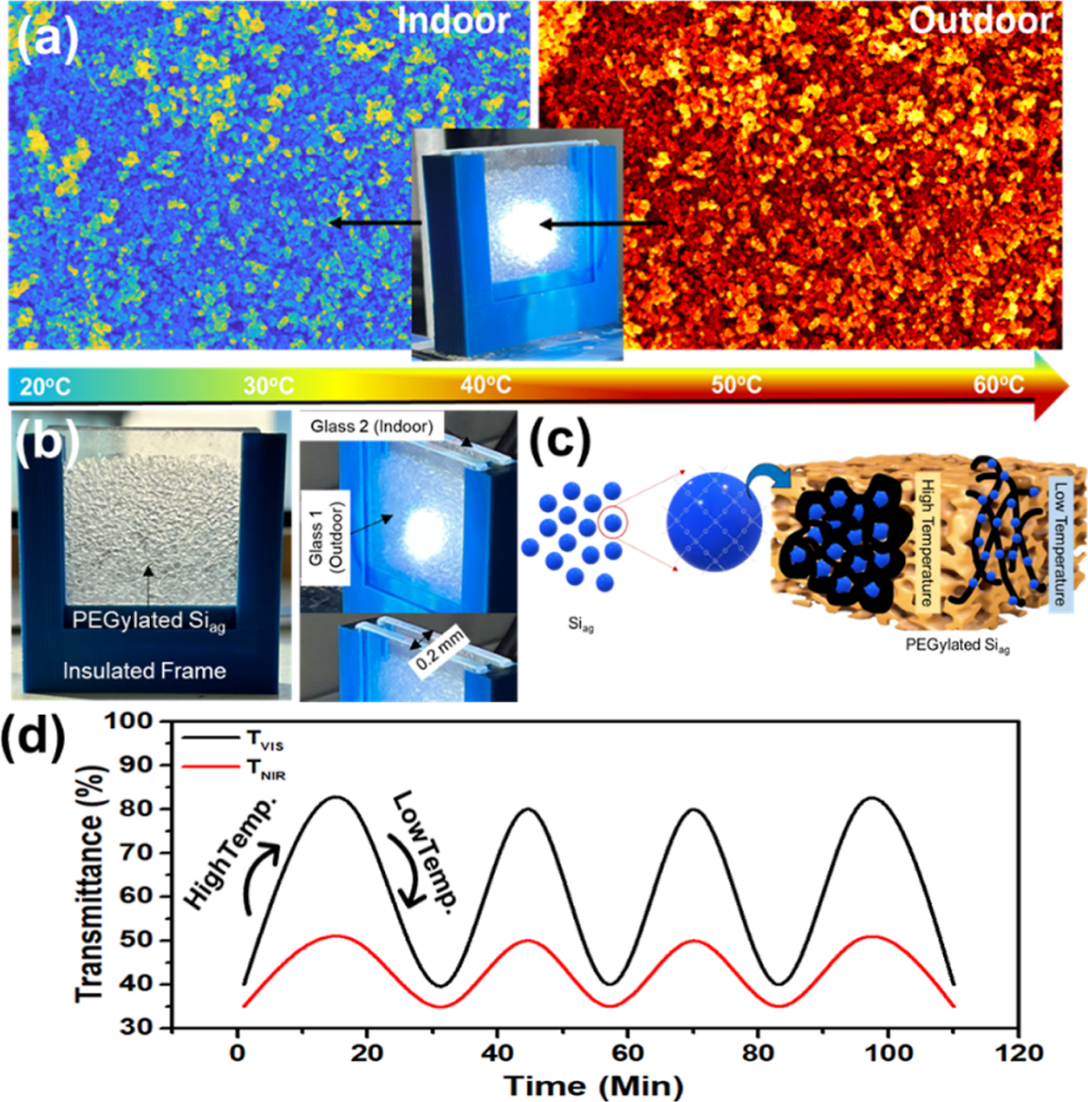
(a) Microstructural SEM thermal mapping, (b) a photograph of the smart window sample containing Si_ag_@PEG-5%, (c) a schematic illustration of the structural mechanism of the Si_ag_@PEG-5% composite sample during its thermal treatment, and (d) the corresponding thermo-kinetic cycle plot at the maximum *T*_VIS_ and minimum *T*_NIR_.

Before the thermal performance of the Si_ag_@PEG composite was evaluated for building applications, a prototype window model was used to assess the initial thermal performance of the Si_ag_@PEG-5% composite sample. The thermal mapping of the Si_ag_@PEG-5% composite in a smart composite window is presented in Figure 5a. The outdoor temperature reached approximately 60 °C, while the indoor temperature remained at approximately 28 °C, demonstrating a good thermal comfort performance. The thermal mapping exhibited various colors at different locations due to the random distribution of the Siag particles on the PEG surface. The extent of cross-linking and network connectivity of the composite through the atomic lattice influences heat conduction, caused by the excitation of vibrational energy levels of interatomic bonds or free electron transport under a thermal gradient.

Figure 5b depicts the smart window prototype utilizing the Si_ag_@PEG-5% composite sample, additionally, the role of PEG in the composite is illustrated through a schematic diagram, as depicted in Figure 5c. It is anticipated that in the PEG framework, the presence of the hydroxyl (−OH) moiety facilitates adsorption of water vapor from diverse humidity conditions. Given that both the composite and Si_ag_ exhibit analogous surface areas, it is postulated that the PEG remains encapsulated within the aerogel matrix. Consequently, even during its phase transition, PEG does not egress from the composite, as depicted in Figure 5c. Importantly, the pore diameter of the Si_ag_, found at 8.56 nm, does not provide sufficient spatial allowance for the unrestrained rotational movement of PEG. However, partial thermal motion of PEG was modestly constrained within this nanoscale dimension (porous confinement), particularly since the free rotation diameter of PEG measures 4.48 nm. In conditions of reduced temperature and due to hierarchical self-assembly mechanisms under ambient conditions, the composite exhibits a marginal translucency. Contrarily, at elevated temperatures, the melting of PEG occurs, but it remains confined within the aerogel microstructure. Therefore, the unique thermodynamic behavior in our experiment may first be attributed to the size confinement effect of SiO_2_ framework on the phase change of PEG and consequent heightened transparency.^40^ It indicates that the supporting SiO_2_ net restricts the liquid leakage of PEG and damages the crystal structure of PEG in the cooling process. The foundational principle of this composite can be likened to a cage designed to preclude leakage or mass loss during cyclic thermal exposure. In addition, the SiO_2_ framework also inhibited the sliding motion of polymer chains outside the SiO_2_ framework, which thereafter inhibited the perfect crystallization of PEG. The hydrogen bonds formed between PEG and SiO_2_ particles serve as cross-linking points, and the composite matrix evenly binds a significant number of SiO_2_ particles to the pore wall surface of the aerogel. The small apertures in the microstructure create numerous intricate porous channels that are suitable for prolonging the gaseous heat transfer pathway, generating multiple phonon scattering at the aerogel interface, ultimately reducing gaseous thermal conductivity and providing improved indoor thermal comfort.^41,42^ Therefore, porous confinement not only alleviates the limitations of PCMs but also enhances their thermo-physical attributes.

Figure 5d displays the thermo–kinetic cycle analysis as a function of time for the maximum *T*_VIS_ and minimum *T*_NIR_, according to CIE photopic luminous human eye efficiency, as shown in Figure 5d. The transmittance exhibited a sinusoidal trend as a function of the temperature and time. The *T*_VIS_ characteristics were modulated within ∼80–40% of transmittance, while *T*_NIR_ exhibits a tuned transmittance of 20–50%, which is relatively shorter than the *T*_VIS_ range. This result suggests that temperature-dependent transparency tuning occurs across different wavelengths. The transparency alteration of the composite was repetitive and recorded for up to 110 min.

### 3.5 Surface Wettability Study

**Figure 6:**
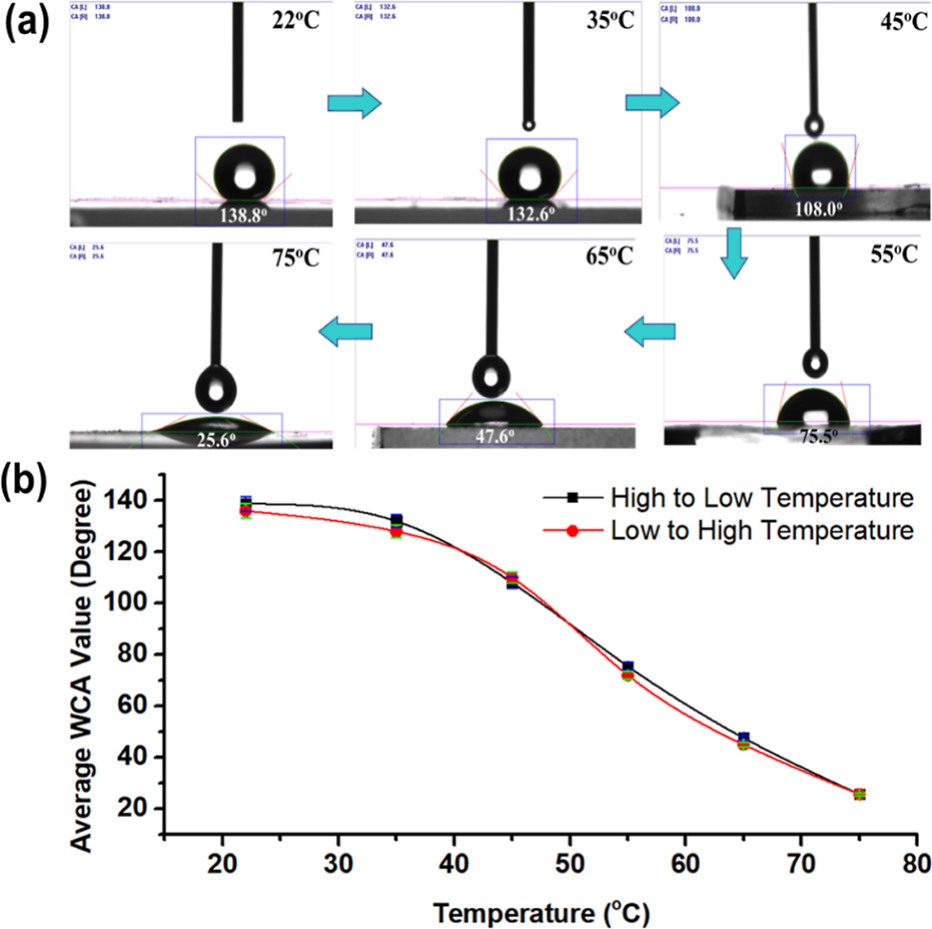
Photographs showing the temperature-dependent water contact angle of the Si_ag_@PEG-5% composite are presented in (a), while (b) illustrates the corresponding plot. The plot indicates that the composite exhibits a switchable surface wettability behavior in response to temperature changes.

The hydroxyl moiety (−OH) inherent to PEG augments water vapor adsorption under varied humidity conditions. Concurrently, surface wettability emerges as a pivotal parameter in preserving the composite’s thermal efficacy. The results of the water contact angle (WCA) test (Figure 6a) indicate that the Si_ag_@PEG-5% composite possesses the advantage of tuning its WCA values from highly hydrophobic to hydrophilic by adjusting the temperature. Notably, despite the inherent hydrophilicity of the precursors, the produced aerogels were rendered hydrophobic merely by altering their geometric conformation. At lower temperatures (<40 °C), the composite displayed hydrophobic characteristics, with a WCA between 138 and 108°, owing to its dense matrix appearance. However, as the temperature increased to 55°, the WCA value reduced to 75° (moderate hydrophilicity), further decreasing to 47°, resulting in hydrophilic characteristics when the temperature reached 65°. Additionally, at higher temperatures (75°), the WCA value tended to become more hydrophilic, with the lowest WCA value of ∼25°. The increase in treated temperature is believed to reduce the composite’s porosity, which may also be due to the phase change of PEG that melts at higher temperatures, resulting in denser pores, higher thermal conductivity, and hence hydrophilicity.

Interestingly, the composite’s tunable wettability characteristic displays a switchable trend with changing temperatures from lower to higher and vice versa, as demonstrated in Figure 6b. Surface morphology is a critical factor that influences these characteristics. This may be attributed to the phase-change heat-transfer surface of PEG, which alters the surface wettability of the Si_ag_@PEG composite between hydrophobicity and hydrophilicity through thermal treatment.^43,44^

The temperature-dependent transparency of smart windows is an important characteristic that enables them to optimize the energy efficiency and indoor comfort. One of the major challenges in producing aerogel windows is the inability to simultaneously achieve high levels of thermal insulation and transparency.

### 3.6 Thermal Conductivity and Heat Capacity Measurement Analysis

**Figure 7:**
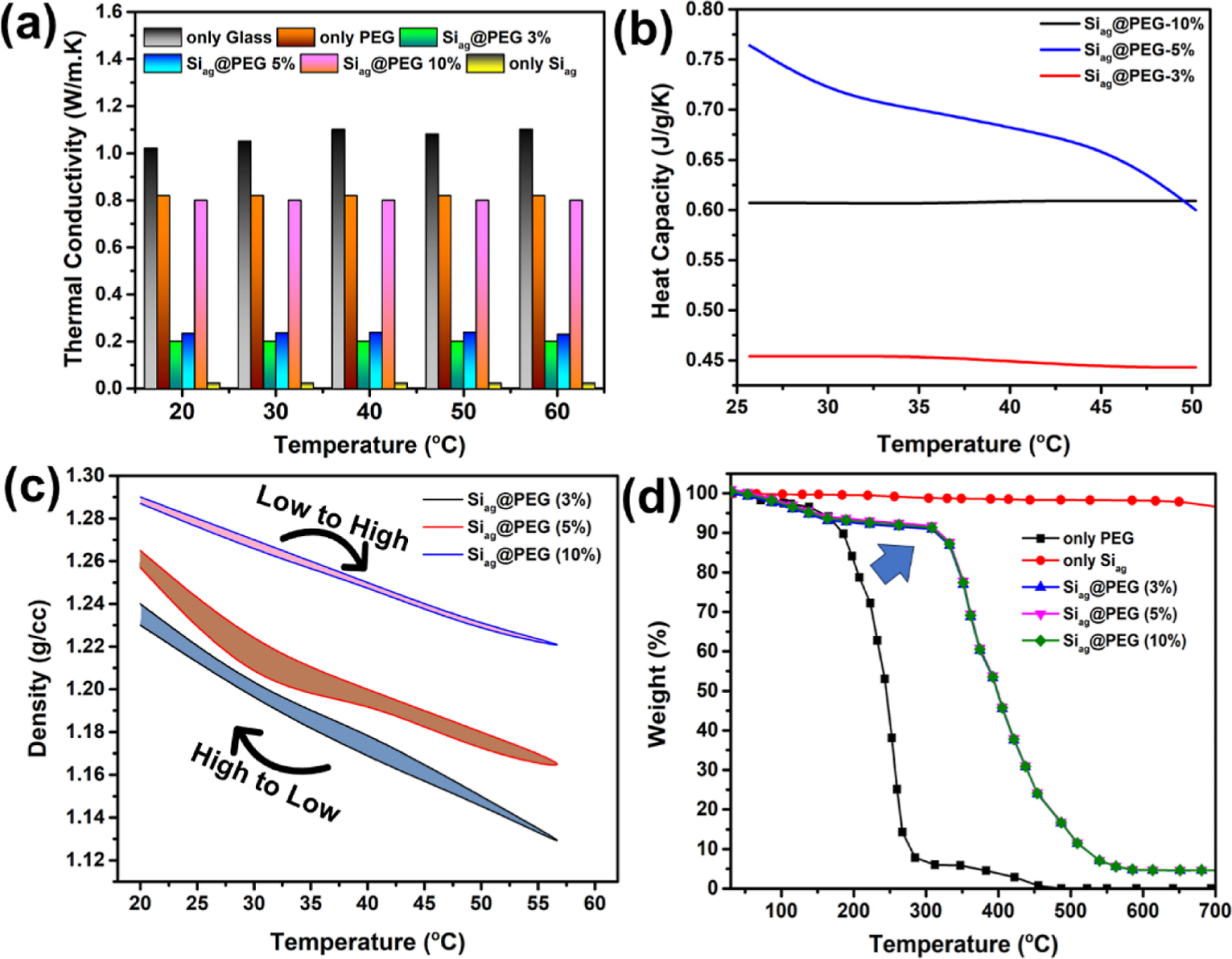
(a) The comparison of the thermal conductivity measurements of all composite samples with their counterparts as a function of temperature, (b) the heat capacity measurements of the composite samples as a function of temperature, (c) temperature-dependent density measurements, and (d) thermogravimetric analysis of the composite samples.

Developing composites with optimized thermal conductivity and heat storage properties is crucial to achieving thermal comfort in various applications. Therefore, the thermal conductivity and heat capacity of Si_ag_@PEG composites were investigated across a temperature range of 20 to 60 °C, as illustrated in Figure 7. The results demonstrated significant variations in thermal conductivity with the Si_ag_@PEG-5% composite exhibiting the lowest value of 0.23 W/m·K among the different compositions. The decrease in the PEG content corresponded to lower thermal conductivity due to the higher proportion of Si_ag_, known for its exceptionally low thermal conductivity (0.023 W/m·K). Conversely, an increase in PEG content led to a gradual increase in thermal conductivity, reaching a maximum of 0.82 W/m·K for the Si_ag_@PEG 10% sample, comparable to that of pure PEG. The increase in thermal conductivity of the composite compared to only Si_ag_ could be considered as significant for thermal energy storage applications. Importantly, all of the thermal conductivity measurements were lower than those of pure glass and pure PEG, indicating the successful reduction in thermal conductivity achieved by the composite. These composites hold promise for thermal comfort applications in windows. Furthermore, the thermal conductivity values remained relatively constant within the temperature range of 20 to 60 °C, indicating the stability of the composite even at elevated temperatures (Figure 7a).

To investigate the effect of temperature on the composite’s heat storage capacity, heat capacity measurements were conducted. Remarkably, the heat capacity remained nearly constant at 0.45 J/g·K for the Si_ag_@PEG 3% composite across the temperature range. As the PEG content increased, the heat capacity also increased, reaching 0.76 J/g·K for the Si_ag_@PEG-5% sample. However, for the Si_ag_@PEG 10% composite, the heat capacity decreased to 0.61 J/g·K, possibly due to PEG agglomeration and a compromised ability of the silica aerogel to retain heat. To be more specific, the composite PCMs material can effectively insulate heat using silica aerogel first and then absorb heat using PCMs owing to the low thermal conductivity of silica aerogel and high latent heat of PCMs. The thermal conductivity of silica-based composite PCMs is lower than that of pure PCMs (Figure 7a), indicating that silica aerogels can effectively suppress the thermal conductivity of composite PCMs and avoid rapid heating.

Additionally, noticeable decrements in heat capacity were observed >35 °C (Figure 7b). It is worth noting that the macroscopic heat release process was slower than the heat storage process. The PEG-aerogel composites successfully addressed concerns related to leakage, stability, and low thermal conductivity. The combination of PEG’s low thermal conductivity and the contact thermal resistance between metal oxide nanoparticles resulted in a significant reduction in surface temperature and notable temperature differentials under solar simulator testing. Temperature-dependent density measurements for various composites were conducted, as illustrated in Figure 7c. The data suggest that the Siag@PEG composites possess a density oscillating between 1.29 and 1.14 g/cm^3^, positioning it within the realm of lightweight wall materials. These measurements indicate that the density variations of the composite, attributable to PEG’s phase change characteristics, exhibit nearly reversible behavior with an average deviation of ±0.2%. This also underscores the material’s stability throughout the thermal treatment. Notably, an increased degree of PEGylation correlates with a heightened density of the composite. The temperature-dependent reversibility pertaining to porosity across the evaluated temperature spectrum further accentuates the intrinsic characteristics of these materials. This confirms the mechanism we predicted for their moisture content and further validates the stability of the composites across a broad operating temperature. The thermogravimetric behavior of the Si_ag_@PEG composites was investigated via TGA, as delineated in Figure 7d. Si_ag_ exhibits commendable thermal resilience, manifesting a negligible weight diminution when scrutinized up to 700 °C. In contrast, when evaluating the only PEG sample, an initial mass reduction at temperatures below 100 °C is ascribed to water evaporation. At an approximate threshold of 208 °C, cellulose fibers of the control film initiate their degradation phase. A conspicuous mass decrement, closely observed around 300 °C, can be attributed to the pyrolytic breakdown of organic moieties, most notably, the cleavage of PEG polymeric chains. Concurrently, the composite manifests superior thermogravimetric stability relative to unadulterated PEG. This suggests that the PEG might be sequestered amidst its phase transitions by the silica aerogel, culminating in enhanced thermal stability by approximately 50%. The composite film, meticulously quantified during its transparent phase, exhibited no discernible weight reduction at <100 °C, signifying an absence of PEG exudation. This investigation substantiates the capability to synthesize a leakage-immune and stable composite when amalgamating PEG, thereby paving the way for the development of multifunctional, energy-optimized intelligent fenestration systems. Furthermore, the PEGylation of silica aerogel augments thermal resilience, with an optimized sample at 5% demonstrating marked improvement. Intriguingly, as discerned from Figure 7d, the weight loss phenomena for all of the composites are monophasic. Collectively, these outcomes corroborate that the Si_ag_@PEG composites possess robust thermal stability, thereby offering a vast operational temperature bandwidth. Such a composite, with a decomposition temperature significantly surpassing its operational range, is propitious for applications in low-temperature heat storage or insulation.

### 3.7 Temperature Distribution of Si_ag_@PEG Composite Filled within Window Glass

**Figure 8:**
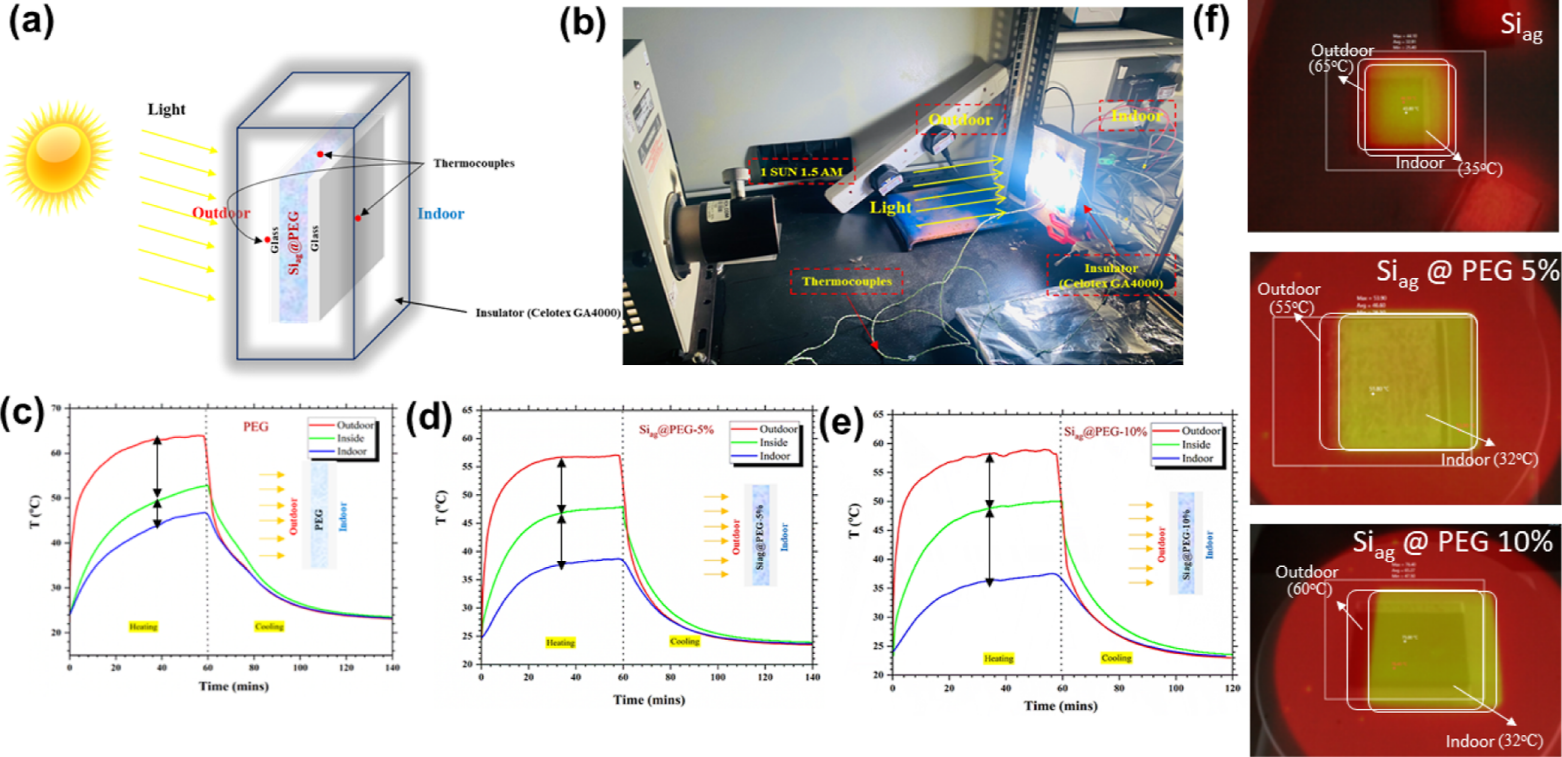
(a) A diagrammatic representation of the experimental setup for temperature profile measurement is presented. (b) The photograph of the experimental setup, which includes Si_ag_@PEG filled window glass setup, connected to thermocouples and a data logger, is shown. (c–e) time–temperature distribution for Si_ag_@PEG, Si_ag_@PEG-5% composite, and Si_ag_@PEG-10% composite, and (f) corresponding IR thermal images, respectively.

In this study, we investigated the real-time temperature distribution of composites made from Si_ag_@PEG, which were filled within window glass and subjected to 1 SUN 1.5 AM operating conditions. The experimental setup for measuring the overall temperature involved a testing process under both heating and cooling conditions, as illustrated in Figure 8a. The surface temperature of the glass was measured by using K-type thermocouples located 5 mm away from the glass surface for both outdoor and indoor operating conditions. These thermocouples^45^ were insulated with a 5 cm thick layer of Celotex GA4000 insulation. Furthermore, an additional thermocouple was inserted inside the outdoor and indoor glasses to measure the temperature of the filled materials, including PEG, Si_ag_@PEG-5% composite, and Si_ag_@PEG-10% composite. The photographic setup used to conduct the thermal performance analysis of PEGylated silica aerogel filled composites is presented in Figure 8b. Continuous illumination was achieved through the use of a sun simulator, facilitating the study of the glazing under extreme conditions. Notably, the spectrum of this simulator closely matches that of natural sunlight, encompassing a range from 250 to 3000 nm. Therefore, the indoor experiment allowed for precise measurements of the device under controlled environmental conditions and a consistent level of irradiation.

The experiments were conducted in two stages. The first stage involved a continuous 60 min exposure to 1 SUN for heating, while the second stage involved a 140 min cooling process without any light exposure. In the presence of light during the heating process, the outdoor temperature, which was in direct contact with the incident rays, increased rapidly. However, in the absence of sunlight, the surface temperature quickly dropped to the ambient room temperature of about 22.0 °C. During the heating and cooling processes, the transparency of the Si_ag_@PEG composites increased (up to 85%) while the translucency decreased (up to 45%).

For PEG-filled composites, higher outdoor, inside, and indoor temperatures of approximately 64.0, 52.0, and 44.5 °C, respectively, were recorded after 60 min of heating (Figure 8c). Once the light was turned off, a rapid decrease in all temperatures was observed. In the case of Si_ag_@PEG-5% (Figure 8d) and Si_ag_@PEG-10% (Figure 8e) composites, lower outdoor, inside, and indoor temperatures were recorded, indicating that an average thermal comfort temperature was maintained with Si_ag_@PEG-based composites.

With 5 wt % loading of Si_ag_, the average temperatures of approximately 55.0, 45.0, and 35.0 °C were recorded at outdoor, inside, and indoor points, respectively. However, little variation in temperature profiles was observed for all points in the case of Si_ag_@PEG-10%, which indicates that window glasses filled with Si_ag_@PEG-5% have optimum thermal performance and maximum temperature difference, making them preferable for building window applications.

Figure 8c–e reveals the effect of PEG during the heating mode. The temperature remained constant after about 20 min, owing to the phase-transition characteristics of PEG. The heat from sunlight was absorbed within the PEG due to its high latent heat of fusion, and almost a constant temperature was achieved until 60 min. Therefore, the incorporation of PEG and Si_ag_ into the window glass enhances the heat-absorbing ability while maintaining transparency, resulting in multifunctionality and energy efficiency properties.

The analyzed thermal topographical images of the composite-coated glass exhibit a substantial decrease in the indoor temperature of up to approximately 32 °C, while the outside temperature increases to 65 °C, as depicted in Figure 8f. Corresponding temperature profiles corroborate these findings, supporting the efficacy of the composite as a viable solution for windows in terms of enhancing thermal comfort when compared to aerogel-based glass alone.

When the smart composite is exposed to heat, from either solar radiation or other sources, the PCM component within the composite absorbs and stores the excess thermal energy. This absorption process prevents the heat from penetrating through the composite, thereby minimizing the heat gain. During periods of lower temperatures or when the surrounding environment requires additional warmth, the PCM undergoes a phase transition, releasing stored thermal energy. This heat release process ensures a balanced and comfortable temperature within the vicinity.

The combined action of the PCM and silica aerogel components within the smart composite allowed for effective heat management. It enables the composite to absorb, store, and release thermal energy as required, promoting optimal thermal comfort while minimizing the reliance on external heating or cooling systems.

Overall, the heat transfer mechanism of the smart composite intelligently utilizes the properties of PCMs and silica aerogels to regulate heat flow, resulting in improved thermal comfort and energy efficiency.

**Figure 9:**
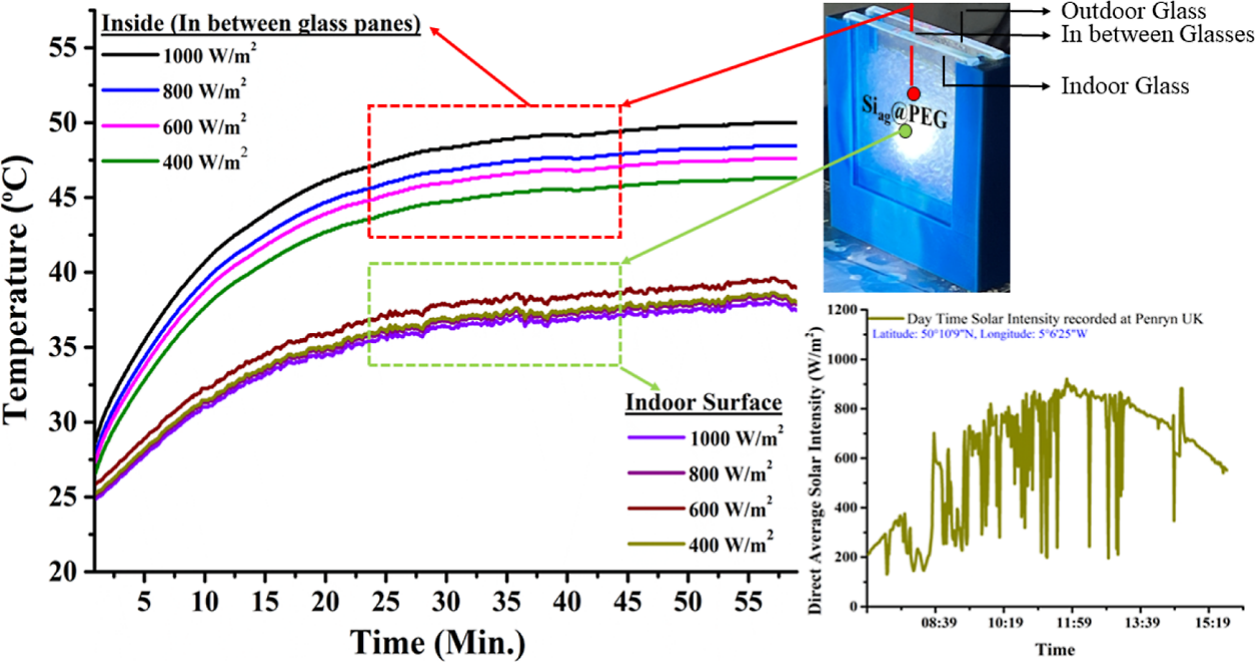
Temperature profile of the Si_ag_@PEG-5% composite under various solar intensities, representing real-time solar irradiance recorded in Penryn, UK, over a day period.

Furthermore, the indoor thermal comfort behavior of the Si_ag_@PEG-5% composite when integrated into a window was systematically evaluated across varying solar intensities, as delineated in Figure 9. The solar radiation intensity was modulated, ranging from a standard value of 1000 to 400 W/m^2^, simulating the diurnal solar radiation observed in Penryn, UK, from 7:00 am to 5:00 pm. To emulate these outdoor conditions in our indoor measurements, we employed a AAA continuous solar simulator, wherein the incoming solar radiation was adjusted between 1000 and 400 W/m^2^. Notably, while the composite’s temperature displayed proportionality to the solar radiation intensity, the indoor surface temperature remained relatively constant, irrespective of these intensities. This suggests a consistent maintenance of thermal comfort levels indoors throughout the day, in contrast to external temperature fluctuations.

This study presents a novel approach for developing aerogel composites with temperature-dependent transparency and surface wetting performance, which are crucial for enhancing indoor thermal comfort levels when they are used in windows. When the composite is exposed to high temperatures, the PCM absorbs heat, preventing it from entering the building. This reduces the reliance on heating, ventilation, and air conditioning (HVAC) systems, leading to energy savings. In comparison to recently reported aerogel composites (Table 1), this approach successfully addresses the challenge of maintaining these key parameters. The composite can be customized to suit different requirements by adjusting the types and concentrations of PCMs and silica aerogel. This flexibility allows for the optimization of the thermal performance based on specific climate conditions and building designs. In addition, the shape-stabilizing properties of the composite, coupled with its processing through various thermal and wettability cyclic performances, suggest the possibility of reversible crack creation. This approach, however, has resulted in a limited thermal performance of the composite. To address this in future studies, a new design incorporating a kirigami-inspired durable structure^46,47^ could be developed using these energy-saving composites.

**Table 1:** Comparative Study of Recent Development in Aerogel-Based Composite Materials for Building Thermal Comfort

sr. no	composite	feature	Reference
1	CaCl_2_·6H_2_O–silica aerogel	physical blending method was adopted	Zhou et al^11^
		structural and thermal properties were investigated	
		reduced thermal conductivity, prevented the leakage, and lessened the supercooling	
2	xonotlite–silica aerogel ceramic fiber–silica aerogel	thermal radiative heat transfer was measured at different wavelengths	Wei et al^42^
		measured the specific spectral extinction coefficient and specific Rosseland mean extinction coefficient	
		reported that radiative conductivities were proportional to the cube of temperature and decreased with the sample density increased	
3	PEG2000–silica aerogel octadecanol–silica aerogel	in situ one-step synthesis strategy	Liu et al^48^
		structural and thermal properties were investigated	
		reduced thermal conductivity, good hydrophobicity, and high latent heat	
4	expanded glass–silica aerogel	mechanical and structural testing	Adhikary et al^49^
		influence of MWCNTs influenced the compressive strength	
5	cement–silica aerogel	aerogel was synthesized from rice husk ash	Abbas et al^50^
		structural and thermal properties were studied	
		density, thermal conductivity and compressive strength were decreased	
6	erythritol–silica aerogel	two-step sol–gel method was used to synthesize the composite	Xiangfa et al^51^
		structural and thermal properties were investigated	
		high heat storage capacity was obtained	
7	polyurethane–paraffin–silica aerogel	two-step method	Yin et al^52^
		morphology and thermal properties were characterized	
		ANSYS 17.0 was used for simulation	
8	palmitic acid–octadecanol–silica aerogel	morphology and thermal properties were characterized	Huang et al^53^
		density functional theory (DFT) carried out for pore size distribution	
		experimental setup for light-to-heat conversion	
9	expanded graphite–paraffin–silica aerogel	two-step method	Huang et al^54^
		morphology, thermal properties, and leakage testing	
		battery module testing experimentally	
10	Al–Si–Al_2_O_3_–silica aerogel	boehmite-coating method followed by heat treatment	Pang et al^55^
		morphology, thermal properties, and transmittance testing	
		thermal insulation and heat storage testing	
11	silica aerogel–polyethylene glycol	silica aerogel synthesized without supercritical fluid drying	current work
		temperature dependent infrared and visible transparency	
		temperature dependent switchable surface wettability	
		maintained a high indoor thermal comfort for day and night	
		COMSOL modeling adapted	

### 3.8 COMSOL Thermal Modeling Analysis

**Figure 10:**
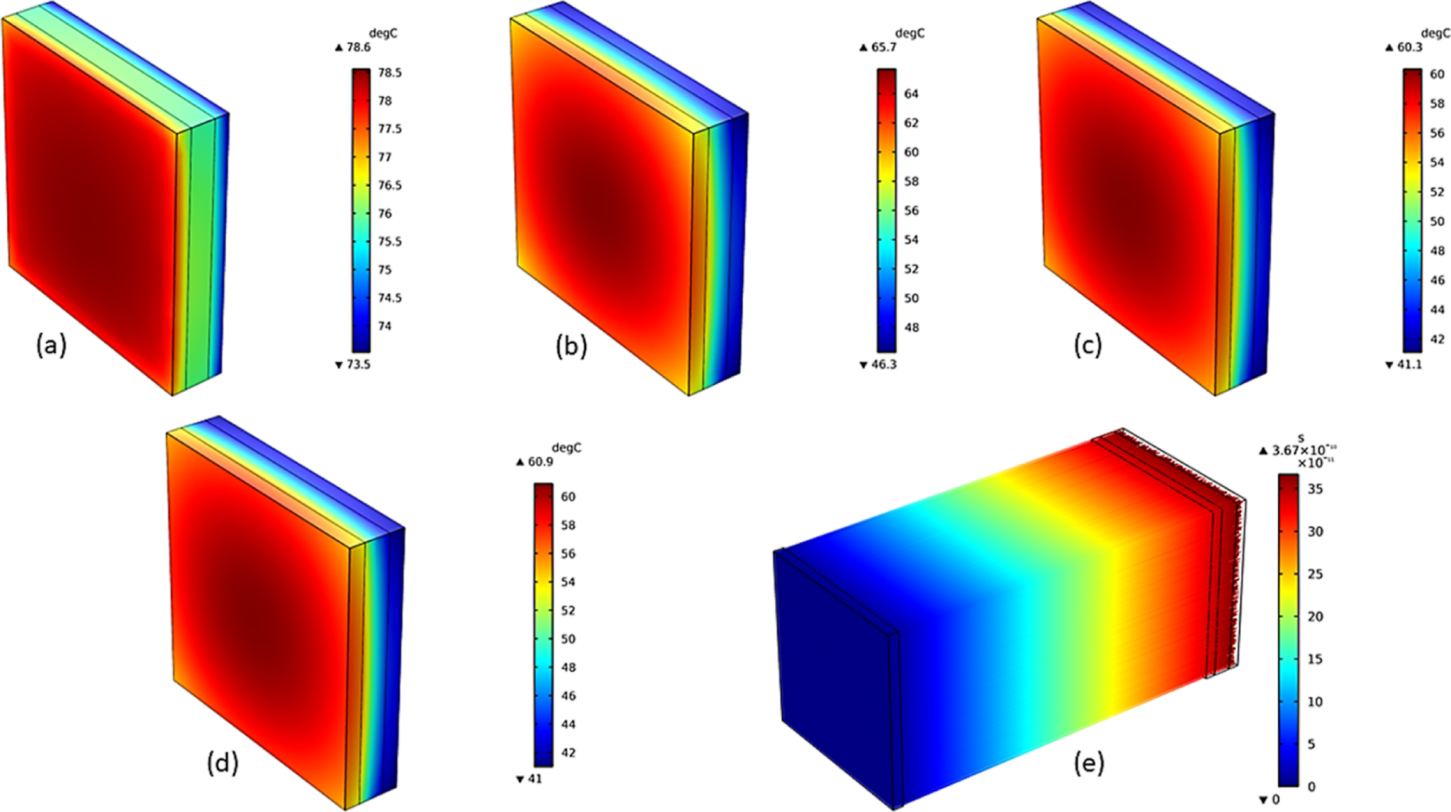
Temperature contours were generated using COMSOL Multiphysics for window glass filled with different materials: (a) air, (b) polyethylene glycol (PEG), composite samples of (c) Si_ag_@PEG-5%, (d) Si_ag_@PEG-10%, respectively, and (e) Si_ag_@PEG-5%, sample’s ray trajectory profile measured in nanoseconds.

We conducted a simulation study to compare and understand thermal comfort levels from the experimental results. Using temperature measurements from ordinary glass and smart composite materials and employing the COMSOL Multiphysics tool, we validated our findings through ray optics and solid heat transfer models. The modeling study’s aim was to ascertain COMSOL’s aptness for such modeling and anticipate future interest in smart composite materials for building thermal comfort. The window glass was filled with air, PEG, Si_ag_@PEG-5%, and Si_ag_@PEG-10% composites, as depicted in Figure 10a–d. The ray tracing was coupled bidirectionally to analyze the phase-change heat transfer phenomenon and the ray optics model under similar operating conditions (1000 W/m^2^ and 1.5 AM). The outputs of the ray optics model were used as a heat source (*Q*_s_) term to solve the heat transfer balance eqs 1–4^56,57^
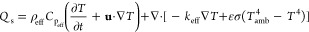
1

The *C*_peff_, *k*_eff_, and ρ_eff_ are defined as follows
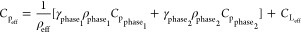
2
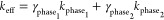
3
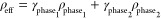
4where, *T*, *C*_peff_, *k*_eff_, ρ_eff_, and *C*_Leff_ are the temperature, effective specific heat capacity, effective thermal conductivity, effective density, and effective latent heat distribution. The γ is the material phase transformation fraction either in phase 1 or phase 2 during melting and solidification processes of Si_ag_@PEG composites, ε is the surface emissivity, σ is the Stefan–Boltzmann constant, *T*_amb_ is the ambient temperature, and **u** is the velocity vector in *x*, *y*, and *z* directions.

In the case of window glass filled with air (Figure 10a), higher temperatures were recorded indoors and outdoors due to the lower resistance of air to heat transfer through the glass as well as its inability to absorb heat from sunlight. The observed outdoor and indoor temperatures in the case of air were approximately 73.5 and 78.5 °C, respectively.

On the other hand, when PEG was filled within the glass (Figure 10b), a significant reduction in outdoor and indoor temperatures of approximately 65.0 and 46.5 °C, respectively, was observed. This can be attributed to the higher latent heat capacity and lower thermal conductivity of PEG, which effectively reduced the total heat transfer rate.

Moreover, the addition of silica aerogel to the PEG composite with 5 wt % (Figure 10c) and 10 wt % (Figure 10d) resulted in a further decrease in outdoor and indoor temperatures. This is due to the effective reduction in thermal conductivity brought about by the silica aerogel. The Si_ag_@PEG-5% composite (Figure 10c) recorded outdoor and indoor temperatures of approximately 60.0 and 41.1 °C, respectively, while only a slight decrease in temperature was observed in the case of the Siag@PEG-10% composite (Figure 10d). The 3D ray trajectories of the incident rays onto the glass surface are depicted in Figure 10e. The balance between ray trajectory and heat transfer is essential for optimal thermal performance, suggesting an interdisciplinary approach for using smart materials in window design.

## 4 Conclusions

In this study, we introduce an advanced solution for enhancing thermal comfort via window integration using a PEGylated silica aerogel-based composite. This composite material was synthesized through a streamlined, economically viable, and energy-efficient method. Exhibiting a noteworthy capability in attenuating near-infrared light, the composite retains a commendable visible light transparency. This ensures a balance between light penetration and thermal comfort. Notably, the composite exhibits tunable wettability, demonstrating a switch in its properties in response to temperature fluctuations, which has profound implications for moisture absorption in building applications. A significant reduction in thermal conductivity, approximately 72%, was observed with the Si_ag_@PEG (5%) composite compared to the benchmarks of pure glass and PEG. These results indicate that the incorporation of PEG and Si_ag_@PEG composites markedly enhances the thermal insulation capacities of windows, resulting in an approximately 20% reduction in indoor and outdoor temperatures, with a differential of about 20 °C. Computational analyses conducted using COMSOL Multiphysics 6.0, incorporating ray optics and heat transfer models, aligned well with our experimental data. This thermal performance can be attributed to the phase change properties of PEG, which allow the formation of a shape-stabilized network where both PEG and silica aerogel disperse heat through their interconnected porous structures. Therefore, the porous confinement strategy of PCMs is a proficient approach to mitigate these limitations while enhancing their thermo-physical attributes. Further, the innovative composite material showcased herein exhibits significant potential as an adaptable and effective solution for multipane windows, skylights, and façade glazing, especially in climatically diverse regions. Comprehensive research into both novel and existing aerogel materials is imperative to discern their application boundaries and to amplify the advantages of their unique properties in the pursuit of more energy-efficient structures. Moreover, the composite’s characteristics of a vast specific surface area, high porosity, and distinct radial-like wrinkled channels make it a suitable matrix for other organic phase change materials, such as paraffin wax, hexadecane, and stearic acid, facilitating the development of shape-stabilized PCMs. In the future, research interest may lean toward the development of smart composite materials for determining thermal comfort levels in buildings. The interplay of ray trajectory and heat transfer modeling is crucial for understanding the exact heat transfer and achievement dynamics. For researchers who integrate experimental data into theoretical building simulations, this presents an interdisciplinary approach to understanding how novel smart composite materials used in glass panes can regulate thermal comfort levels. The work may inspire the future development of multifunctional smart windows and spatiotemporal light control methods.
